# Practical Consequences Resulting from the Analysis of a 21-Multigene Array in the Interdisciplinary Conference of a Breast Cancer Center

**DOI:** 10.1155/2018/2047089

**Published:** 2018-07-10

**Authors:** Hans-Ullrich Voelker, Lea Frey, Annette Strehl, Michael Weigel

**Affiliations:** ^1^Dept. of Pathology, Leopoldina Krankenhaus GmbH, Gustav-Adolf-Str. 8, D-97422 Schweinfurt, Germany; ^2^Institute for Pathology, University of Wuerzburg, Josef-Schneider-Str. 2, D-97080 Wuerzburg, Germany; ^3^Dept. of Gynecology and Obstetrics, Breast Cancer Centre, Leopoldina Krankenhaus GmbH, Gustav-Adolf-Str. 8, D-97422 Schweinfurt, Germany

## Abstract

During the multidisciplinary planning of postoperative therapy after breast cancer, borderline cases can arise with no clear rationale for or against adjuvant chemotherapy. In 50 hormone- receptor-positive, Her2neu-negative carcinomas of the breast with no or only minimal lymph node involvement (max. pT1a) we initiated an Oncotype DX® multigene assay in addition to the evaluation of usual parameters. In the oncology conference a vote for or against chemotherapy was taken on the basis of the conventional criteria for decision-making before the test results were available. The final recommendation was made after the multigene test. In 32 breast carcinomas (64%) a low recurrence score could be documented, while 26 (32%) showed an intermediate RS and 3 (6%) showed a high RS. In most cases the result of the test could validate the choice of therapy established using conventional criteria. In 5 cases the initial recommendation for adjuvant therapy was revised, and in 3 cases chemotherapy was secondarily recommended after evaluation of the test results. Conversely, in some cases a low or intermediate risk constellation did not argue against a recommendation for adjuvant chemotherapy. Altogether, the results of our study do not indicate that a multigene assay should be used as a routine diagnostic tool. Instead a thorough compilation and careful analysis of conventional parameters for therapeutic decision-making should take precedence, with special emphasis on histopathological and immunohistochemical results. In selected cases, however, a multigene assay can be a useful tool in the deliberation for or against a therapeutic pathway.

## 1. Introduction

For risk stratification in breast cancer, conventional parameters such as age at onset, menopausal status, tumour size and stage, histological grading and subtype, node status, resection margins, metastasis, and proliferative activity (Ki67) as well as an evaluation of hemangioinvasion and lymphangioinvasion are used. The determination of expression of hormone-receptors and Her2/neu as prognostic and predictive parameters is indispensable.

Gene expression analyses have been used to define intrinsic subtypes of breast cancer at the molecular level, showing differences in disease progression and treatment response:* luminal A and B, Her2/neu*-positive,* and basal-like* [1, 2]. Since comprehensive genetic testing is not feasible in routine diagnostics due to cost and time reasons, the immunohistochemical surrogate parameters oestrogen receptor (ER), progesterone receptor (PR), Her2/neu, and Ki-67 are used [3, 4]. Under the St.-Gallen-Consensus of 2011, the therapy recommendation is based on the intrinsic subtypes determined in this way. In addition to surgery, chemotherapy is the choice of treatment for triple-negative cancers [5]. For the adjuvant treatment of* luminal A-subtype*, endocrine therapy is often sufficient alone, whereas* luminal B-cancers* are more aggressive. The distinction of luminal carcinomas is also based on the immunohistochemical determination of Ki-67 proliferative activity. Ki67 is a protein that is regularly expressed in the nucleus in the active phases of the cell cycle, but not in the G0 phase [3]. In paraffin-embedded tissue, cells in active cell cycle phases can be visualized with the antibody MIB1 through a positive nuclear stain [6]. However, there is no generally accepted threshold between low and increased proliferative activity, although it is often reported as 14% [7, 8]. A complicating factor is that currently no generally standardized evaluation procedure can be specified and thus the determination is subject to procedural inaccuracies.

Thus, it is quite often difficult to decide in favour of or against adjuvant chemotherapy for luminal breast cancers. Multigene tests on tumour tissue can help to estimate the individual risk for relapse more accurately. Tests available in Germany include Oncotype DX, Mammaprint®, EndoPredict®, and Prosigna® Assay.

Oncotype DX is a 21-gene assay available in Europe since 2009 with a comprehensive database [9, 10]. In a central laboratory in the USA, the standardized and quality-controlled analysis is carried out by quantitative real-time polymerase chain reaction (qRT-PCR). 21 genes are investigated, including 16 cancer-associated genes and 5 reference genes. From the result, the numerical recurrence score (RS) is determined, which reflects a defined risk of recurrence within 10 years from the time of diagnosis. It is divided into three risk groups defined for clinical validation:* low risk* (RS <18), intermediate* risk* (RS 18-30), and high* risk* (RS≥31). The prognostic significance has been shown by a number of studies [11–13]. For patients with* high risk* tumours, the benefits of adjuvant chemotherapy have been demonstrated [14].

However, these tests are costly and are not regularly reimbursed by all health insurance companies. This therefore raises the important question of the actual benefit of multigene testing in everyday clinical practice, regardless of the well-proven benefit with regard to the prognostic value. At the breast cancer center in the Leopoldina Hospital GmbH Schweinfurt, the actual influence on the decision for or against adjuvant chemotherapy was evaluated over a period of three years by comparing therapy recommendations with and without knowledge of the Oncotype DX result.

## 2. Material and Methods

In the period of 2013 to 2016, 954 primary cases of breast cancer were treated at the breast cancer center of the Leopoldina Hospital GmbH and discussed in the weekly interdisciplinary tumour conference. The basis for decision-making for further treatment planning was the S3 guideline valid during this period. For 50 hormone-receptor-positive, Her2/neu negative carcinomas, a 21-multigene test (Oncotype DX) was additionally initiated using tumour tissue. In these selected cases, it was unclear whether or not the affected patients benefited from adjuvant chemotherapy, even after evaluating the overall clinicopathological risk profile.

The included breast carcinomas showed either no lymph node metastases or a low nodal involvement with a maximum stage of pN1a.

All required conventional parameters were known, especially age, menopausal status, tumour size and stage, histological subtype and grade of tumour, number of lymph nodes removed and affected, resection status, coexistent in situ lesion (DCIS/LCIS), and staging results. Tumour differentiation (G1, G2, and G3) was based on the modified combination regimen of Elston/Ellis. The immunohistochemical parameters (Ki-67 index, hormone-receptors, and Her2/neu status) were determined according to current standards. All immunohistochemical stains were carried out automatically using a BondMax® (LEICA) staining system.

For the oestrogen receptor (ER), the commercially available antibody clone 1D5 was used and for the progesterone receptor (PR), the clone PgR363 (both, ER and PR antibodies, from DAKO, Agilent Pathology Solutions, Germany, D-22083 Hamburg) was used. To determine the hormone-receptor status, the immunoreactive score according to Remmele and Stegner (IRS) was used, which is calculated from the percentage of positively stained tumour cells and the staining intensity. The score of 0 counts is negative, 1-3 is weakly positive, 4-6 is moderately positive, and 8-12 is strongly positive. The pathology department of the Leopoldina Hospital Schweinfurt GmbH successfully completed the ring trials with an evaluation procedure (QuIP) during the study period.

The Her2/neu test was carried out immunohistochemically (clone c-erbB-2, DAKO), where the result was ambiguous an additional FISH analysis was performed. The evaluation of the stain was carried out according to S3 guidelines; 10% tumour cells were set as a threshold for the positivity with complete membrane staining. Again, the pathology department successfully participated in the relevant ring trial.

The Ki67 stain (clone MIB-1) was carried out according to a standardized protocol. The percentage of tumour cells with immunohistochemical evidence of MIB1 protein expression was expressed as Ki-67 proliferation index. The staining quality was proven by a standardized annual interlaboratory test.

### 2.1. Multigene Assay

As a supplementary test, Oncotype DX testing was initiated after an interdisciplinary case discussion in the tumour board. The result was generally available seven to ten days after shipment of the sample in form of a written report. A paraffin block containing tumour tissue from the routine processing of the surgical specimen was used for analysis. The dispatch is handled by a logistics service provider who primarily collects all samples in a central pathology department (Optipath, Frankfurt/Main) which is determined by the provider of the Oncotype DX test. From there, the material is shipped to a central laboratory in the USA from which the test results are issued.

### 2.2. Preliminary and Final Therapy Decision

In the interdisciplinary tumour conference a preliminary decision was made as to whether adjuvant chemotherapy would be beneficial and acceptable on the basis of the previously known risk profile. This result was noted in the tumour conference protocol. After receiving the results of the multigene analysis, the discussion was repeated and the test result was incorporated into a definitive therapy recommendation. Only the final decision was communicated to patients.

The ethics committee of the University of Wuerzburg (Versbacher Straße 9, D-97078 Wuerzburg, chairman Professor E.-B. Bröcker, www.ethik-kommission.medizin.uni-wuerzburg.de) does not stipulate a separate consultation or request a special permit for the further retrospective processing of internal data from routine diagnostics. The exclusive use of data from routine clinical examination and treatment as well as the retrospective analysis of this information does not require the informed consent of concerned patients included in this study. In the contract for in-patient treatment at the Leopoldina Hospital all patients give their written consent to the further internal processing of their data. Prerequisite for this is that the data compilation is carried out on the basis of ethical aspects and under strict protection of data privacy. This can be guaranteed by the authors. The data analyzed in this study was exclusively obtained from routine examinations, which were seen as essential for the further treatment of patients and were conducted independently of further retrospective processing of the results. The included patients were completely and irreversibly anonymized after documentation of the relevant parameters (tumour stage, tumour biology, result of Oncotype DX, etc.) was complete, so that it was not possible to trace the data of a single patient.

## 3. Results

### 3.1. Characteristics of the Entire Cohort

The investigated population consisted of women with an average age of 53 ± 11 years. Male breast cancers were excluded. All breast cancers tested were hormone-receptor-positive (oestrogen receptor average 11.1 ± 2, progesterone receptor average 9.8 ± 3.8) and Her2/neu negative (score 0: 34%, score 1+: 64%, and score 2+ with negative FISH analysis: 2%). 66% were node-negative; the remainder showed a maximum of three affected lymph nodes. The Ki67 index was evenly distributed below and above the cited threshold of 14%. Details are given in** Tables 1 and 2**.

### 3.2. Results of the Multigene Test

The mean recurrence score (RS) of the total cohort was 26.7 ± 8.5. The smallest RS was 5; the maximum value was 37. In 32 cases there was a low RS <18 corresponding to a mean 10-year recurrence risk of 6.8%. Sixteen cases showed an RS between 18 and 30, corresponding to a 10-year recurrence risk of 14.3%. Three cases had score values over 30, corresponding to a high risk of recurrent disease (mean 10-year risk 30.5%).

For several conventional parameters such as tumour size (r = -0.13), tumour stage, histological type, and lymphatic and vascular invasion, there was no association with the Oncotype DX risk groups. In the high risk group, for example, there was no case with lymph node or blood vessel invasion; in the low risk group, the tumours showed the largest diameter. Also the nodal status was not significantly linked to the RS: the three* high risk* cases were node-negative; in the* intermediate risk* group, n = 6 (37.5%) were node-positive and n = 10 (62.5%) were node-negative, and in the* low risk* group n = 9 (20%) were node-positive and n = 22 (80%) were node-negative.

### 3.3. Age and Recurrence Score

When looking at the age structure of the three risk groups, the majority in the* low risk* group were younger on average (52 ± 10 years) and in the* high risk* group there were primarily elderly patients (64 ± 6 years). The mean age of the* intermediate risk* group was 54 ± 11 years. The differences to the three* high risk* cases are not significant due to the small number of cases (r = 0.14).

### 3.4. Tumour Differentiation and Recurrence Score

Well-differentiated breast carcinomas (G1, n = 4) were found exclusively in the* low risk* group. Otherwise, a moderately differentiated carcinoma (G2) was seen with low RS (n = 27). In the group with* intermediate risk* 13 carcinomas were moderately differentiated (G2) and three were poorly differentiated (G3). The RS at G2 was between 18 and 29; the G3 tumours showed an RS of 18, 21, and 29. Thus, there was no dependency on grading.

**Figure 1:**
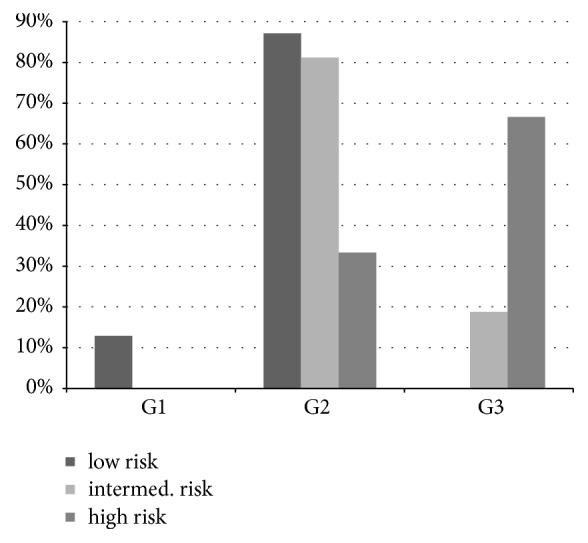
**Histological grade of cancers within the three risk groups** (well differentiated = G1, moderate = G2, and poor = G3).

In the high risk group, two poorly differentiated carcinomas and one G2 carcinoma were found (see also**Figure 1**).

### 3.5. Ki67 Index and Recurrence Score

**Figure 2:**
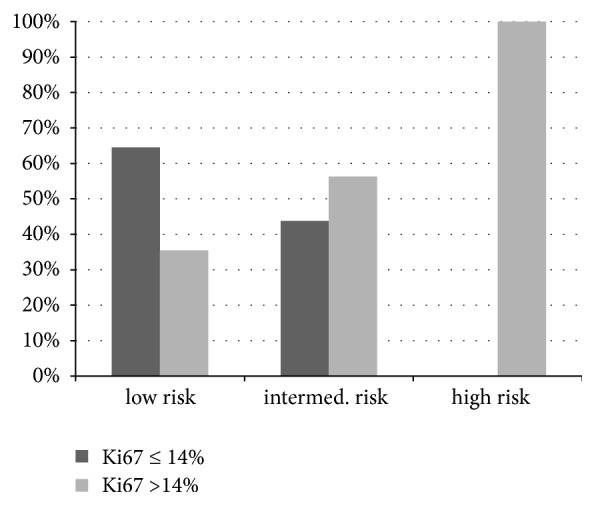
Index of Ki67 (%) within the risk groups.

As the RS increased, there was also an increase in the Ki67 index (r = 0.65). While the mean Ki67 index was 9.3% ± 6.0 in the* low risk* group, it rose to around 17.5%  ± 6.0 in the* intermediate risk* group and 41.7%  ± 8.3 in the* high risk* group. However, the differences are not significant because of the low number of cases, especially in the* high risk* group. In the* low risk* group, nearly two-thirds of cases (n = 20, 64.5%) showed Ki67 values <14% (*luminal A*), with the remaining four cases with a proliferation of > 20%. In the* intermediate risk* group, the Ki67 index was above 14% in nine cases (56.3%), with values over 20% achieved in four of these nine cases. In the* high risk* group, a Ki-67 index of >14% and sometimes >20% (luminal* B) *was noted in all cases (**Figure 2**).

### 3.6. Hormone-Receptor Status and Secondary Control of Immunohistochemistry in the Multigene Test by qRT-PCR

In Oncotype DX testing, the qRT-PCR method is additionally used to determine the RNA expression of the oestrogen and progesterone receptor (ER, PR). All samples tested showed a positive ER status as in the immunohistochemical examination. When comparing the mean of the three risk groups, the highest mean was seen in the* low risk* group (10.3 ± 1.2) and the lowest one in the* high risk* group (9.3 ± 1.8), consistent with data from the immunohistochemical staining. For the PR status determined by qRT-PCR, the highest mean was seen in the* low risk* group (8.6 ± 1.2) similar to the immunohistochemically obtained results. In two cases, a negative PR status was found in the PCR, whereby the immunohistochemistry was weakly positive (IRS 1 each). Two immunohistochemically negative cases were weakly reactive in the PCR. Overall, however, a good consistency of the values was seen. The correlation between RS and receptor expression was r = -0.38 for ER and r = -0.58 for PR.

### 3.7. Test Result and Final Therapy Recommendation

**Figure 3:**
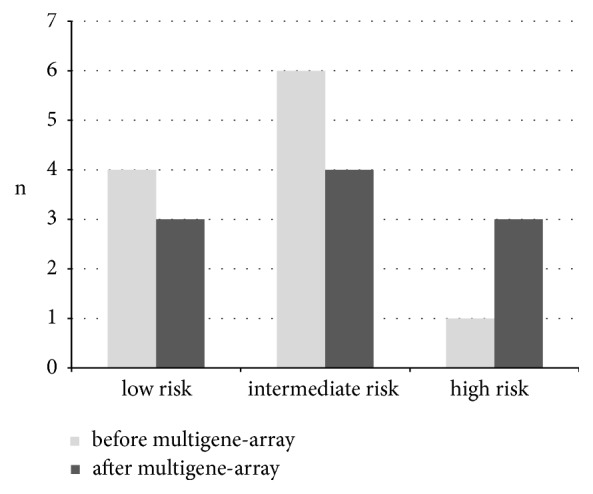
Number of cases with recommendation for adjuvant chemotherapy before and after results of the multigene assay.

Prior to receiving the test result, a decision was made in favour of adjuvant chemotherapy for 12 patients (24%) in the interdisciplinary tumour conference based on the risk profile. After risk stratification by the multigene test, the provisional recommendation was revised in 8 cases of the overall cohort. A secondary decision against chemotherapy was made in five cases where it had initially been supported (**Figure 3**).

In the* low risk* group, four cases received a recommendation in favour of adjuvant chemotherapy prior to testing. After the multigene test, the tumour conference upheld the recommendation for chemotherapy in two cases despite a low RS; an antihormonal monotherapy instead of an initially favoured chemotherapy was decided on in two cases. In one case, adjuvant chemotherapy was recommended instead of the previously favoured antihormonal therapy. Overall an amendment to the provisional decision was made in three cases.

Interestingly, adjuvant chemotherapy was chosen in one case despite a low risk of recurrence following multigene testing. This was a 49-year-old woman with multicentric breast cancer (NST G2) with positive nodal status (isolated metastasis), concomitant high-grade DCIS (extensive disease), and a Ki67 index of 15%. This patient had a high desire for security but nevertheless wanted all the available parameters to be taken into consideration, so that the test was initiated as an additional tool to help in the final ruling. In the end, chemotherapy was recommended despite a* low risk* situation due to a high tumour load (in accordance with S3 guidelines). In spite of having a low RS, two other patients nevertheless received a recommendation for adjuvant chemotherapy: one due to an age at onset of 28 years (pT2, pN0, and Ki67, 15%) and one patient aged 47 (pT1c, pN0, and Ki67, 10%) with an extremely high individual safety need.

In the* intermediate risk* group, adjuvant chemotherapy was favoured in seven cases prior to the multigene test and was then confirmed in four of these cases. Here, above all, the individual circumstances were taken into careful consideration when making the decision. Three G3 carcinomas were also found in this group. For two patients, chemotherapy was not recommended despite poor differentiation (G3) with proliferation rates of max. 15% in Ki67 at an age of 57 and 73 years with many comorbidities. RS values of 18 and 29 supported this decision. For the third patient (71 years, pT2 pN1a (1/12), Ki67 15%, case 20 in Table 4) chemotherapy was primarily recommended. Since this was rejected by the patient, the multigene test was initialized as an additional argument to support or refute the recommendation. With an RS of 21, a decision was then made against adjuvant chemotherapy in accordance with the patient's wishes.

In the group with a high RS, all three patients were given a recommendation for adjuvant chemotherapy. This decision was overturned in 2 cases (**Table 3** shows these numbers again). Both of the patients where the initial vote was against adjuvant chemotherapy had G3 carcinomas (with preoperative tumour biopsies giving a diagnosis of intermediate differentiation G2) and significant comorbidities at the ages of 64 and 69 years, which explains the initial tendency towards a cautious recommendation.

**Table 4** shows the compilation of the eight cases with a modified therapy recommendation.

## 4. Discussion

In breast cancer, adjuvant therapy is often more strongly oriented towards tumour grading and individual tumour biology as well as tumour stage (as defined by the TNM formula) when compared to other tumour entities. In addition, patient age and menopausal status play an important role. Immunohistochemical surrogate parameters (hormone-receptor and Her2/neu status as well as Ki67 index) can be used to subdivide breast carcinomas into five intrinsic (molecular) subtypes. However, with luminal breast cancer (Her2/neu negative, hormone-receptor-positive), it is not always clear which patient will benefit from adjuvant chemotherapy based on the classic clinical and histopathological factors. Against this background, multigene tests such as Oncotype DX have been developed to better assess the individual risk of recurrence. How or whether a multigene test specifically influences therapeutic decisions was examined in this study.

During a period of three years, the treatment plans for 954 patients diagnosed with breast cancer were discussed and confirmed in the interdisciplinary tumour board of our breast cancer center. In 50 tumours, multigene testing was indicated due to difficulties in favouring or rejecting adjuvant chemotherapy. Thus only 5.2% of all cases required this extended analysis. However, the additional examination of the tumour tissue also meant that a final assessment for or against adjuvant chemotherapy usually had to be delayed by one week, sometimes two weeks, until the test result was available. The fact that this waiting period would not be detrimental to further treatment and outcome had to be explained to the affected patients. Nevertheless, uncertainties arose in individual cases among the patients.

The risk groups as indicated by the recurrence score (RS) were unevenly distributed in the 50 cases studied: 31 patients (62%) had* low risk* carcinomas, 16 (32%)* intermediate risk, *and only three patients (6%)* high* risk tumours. However, it was also expected from conventional risk analysis that a* high risk* situation would arise only rarely.

In the comparison of the mean age at diagnosis in the three risk groups, a higher age was observed in the* high risk* group, with the difference not being statistically significant due to the small number of cases in this study. Corresponding to the high significance of tumour differentiation, G1 carcinomas were found only in the low risk group. On the other hand, moderately differentiated carcinomas could be found in all groups and G3 carcinomas in both medium and high risk tumours. Flanagan et al. could show a significant association between RS and tumour differentiation [15]. They concluded that Oncotype DX testing was not required for G1 and G3 tumours since there was a correlation between tumour differentiation and RS in these cases. Despite the small number of cases, we were not able to fully confirm this result, since in our group three G3 carcinomas had only a moderate RS and in some of these cases a vote was made against adjuvant chemotherapy taking into account the sum of all relevant parameters. The explicit wish of the patient also played a significant role in the case of a 71-year-old female patient, despite the fact that chemotherapy was initially recommended based on relevant guidelines. The multigene test was initiated in this case because the patient explicitly refused chemotherapy and another argument was needed to help with better counselling in this personal decision. In two other patients with G3 carcinoma, adjuvant therapy was not voted for under consideration of the comorbidities. Again, the test with intermediate RS helped to justify this therapeutic recommendation.

In the three risk groups we also found an increase in the Ki67 index as the risk increased. It cannot however be inferred whether a high Ki67 value is always accompanied by a high RS. Even with low RS, 35.5% of cases showed Ki67 indexes over 14%. In this context, difficulties in determining the Ki67 index should be noted. There is currently neither a generally accepted or standardized method of testing nor a single threshold that reliably separates a “low” from a “high” Ki67 index. The 14% threshold is derived from the 2011 St. Gallen Consensus, which uses this cut-off value to differentiate the intrinsic* luminal A* from* luminal B-*subtype. Despite the difficulties of determining Ki67 (marked dependence on the degree of fixation and preservation of the tissue, the used antibody, the dilution, and the investigator), Allison et al. showed a significant association with the resulting RS (p = 0.007) for Ki67 as the only proliferation and cell cycle markers investigated [16]. Based on the other parameters included in the investigation, the authors concluded that a combination of pathologically determined parameters could be used in the estimation of the expected RS.

As expected, the mean scores for the ER and PR hormone-receptors in the group studied dropped from the* low risk* group to the* high risk* group. The decrease of the percentage of strongly positive values for PR was particularly clear in comparison. In two cases with a high RS, a negative PR score was observed. Allison et al. demonstrate that PR expression has a greater value for the prediction of the RS than ER [17]. Also the data published by Tang et al. suggests a strong correlation between high RS and low PR scores [17], just as Clark et al. found an inverse relationship between PR expressions measured by modified H-score and RS, which was independent of the Nottingham Grading [18]. They conclude that a special focus on PR scores can help to identify patients that may profit from additional multigene testing.

The concordant data of receptor status in immunohistochemistry and Oncotype DX lead to successful efforts for a prediction of RS from conventional parameters. Klein et al. describe a model by linear regression analysis in which a prediction of Oncotype DX-RS becomes possible on the basis of Nottingham Grading and immunohistochemical analysis of ER and PR as well as Her2/neu, tumour size and Ki67-percentage [19]. This new Magee-equation (http://path.upmc.edu/onlineTools/mageeequations.html, accessed June 5th 2018) shows a high concordance with original Magee-equation and its use can diminish the need of multigene testing in breast cancer. Furthermore, Magee 3 equation could predict the response of ER-positive, Her2/neu negative breast cancers in neoadjuvant concepts [20]. Cuzick et al. and Sheri et al. described the use of IHC4 (values by immunohistochemistry of ER, PR, Her2/neu, and Ki67) with similar predictive intention in early breast cancer and neoadjuvant concepts [21, 22]. The IHC4-score could also be helpful for estimating the result of Oncotype DX and lead to using expensive multigene testing only in cases with strict medical indication.

The main focus of the present observational study was to examine how the result of the multigene test actually influences the therapeutic recommendation for or against adjuvant chemotherapy in the daily practice of a breast cancer center. To our knowledge, it has seldom been explored explicitly unlike the well-described general prognostic value of multigene arrays. In our cohort, the use of the test changed the treatment decision in only eight cases. This corresponds to 16% of the tumours tested and only 0.8% of all breast cancers treated during this period. In comparison with the literature, this is a low rate of modification. Comparable studies showed values between 24.9% and 32.0% [23, 24]. It must be noted that there was a greater initial tendency towards adjuvant chemotherapy in these studies than in our tumour center. It is also fitting that a decision against adjuvant chemotherapy is made more frequently after the test, corresponding to the literature [25]. Overall, it has been shown that a carefully considered treatment recommendation based on the classic clinical and histopathological factors is supported by the results of multigene testing in most cases. The relatively rare occurrence of a* high risk* situation observed by other authors leads to the correction of a preliminary decision to chemotherapy in only a few cases [26].

In no case was the result of the multigene test the sole determinant for a definitive therapeutic decision. Despite a* low risk* situation, adjuvant chemotherapy sometimes had to be recommended in the final meeting due to the overall constellation (for example, a patient with high safety needs). In the group with* intermediate risk*, the definitive therapeutic decision was still heavily dependent on parameters such as age or tumour size. In other studies adjuvant chemotherapy was performed in only 2.0% to 7.7% of* low risk* cases while in the* intermediate grade* group it was performed in just over a third of the cases. In the* high risk* group, the rate was 80.8% in one report. Here decisions were made against adjuvant chemotherapy due to the overall situation despite a high RS [27].

## 5. Conclusion

Genetic tests on tumour tissue allow an in-depth insight into tumour biology but cannot be the means of entry into* personalised medicine *for cost reasons in the clinical routine of a breast cancer center. The small number of tests that are necessary and the resulting low rate of modification in therapeutic decisions impressively demonstrate the importance of careful analysis of all conventional parameters of breast cancer. However, in selected individual cases multigene tests provide important supplementary information on tumour behaviour and are helpful in final decision-making.

## Figures and Tables

**Table 1:** **Subtypes and stages of included cases of breast cancer.** NST = invasive breast cancer of no special type (NST). 17 cases with N+ showed one metastasis in n=10, two metastases in n=6, and three metastases in only one case.

	**n**
**Subtype of tumor**	
invasive NST	44
invasive lobular	5
other types	1

**Grading**	
G1	4
G2	41
G3	5

**T-Stage**	
pT1b	2
pT1c	25
pT2	22
pT3	1

**Lymphonodal stage**	
nodal negative	33
nodal positive	17

**Lymphangioinvasion**	22

**Hemangioinvasion**	2

**Table 2:** Immunoreactive score (IRS) of oestrogen and progesterone receptor (ER and PR) and index of Ki67 (%).

	**n**
**IRS Score ER**	
0 negative	0
1 – 3 weakly positive	1
4 – 6 moderately positive	4
8 – 12 strongly positive	45

**IRS Score PR**	
0 negative	2
1 – 3 weakly positive	3
4 – 6 moderately positive	5
8 – 12 strongly positive	40

**Ki67-Index**	
Ki67-Index <14%	28
Ki67-Index >14%	22

**Table 3:** **Modified recommendation for therapy after results of Oncotype DX** (CT = adjuvant chemotherapy; AHT = antihormonal therapy).

	low risk n	intermediate risk n	high risk n	Total

	31 (62%)	16 (32%)	3 (6%)	

**Change of recommendation**				

CT->AHT	2	3	0	5

AHT->CT	1	0	2	3

Total	3	3	2	8

**No change of recommendation**				

CT->CT	2	4	1	7

AHT->AHT	26	9	0	35

Total	28	13	1	42

**Table 4:** Details for cases with change of recommendation. ER/PR IRS: immunoreactive score for hormone receptors; RS: recurrence score; CT: adjuvant chemotherapy.

**Case no. **	**Age**	**pT**	**pN**	**L**	**V**	**G**	**ER IRS**	**PR IRS**	**Ki67 %**	**RS**	**before Oncotype**	**after Oncotype**
6	56	2	0 (0/2sn)	1	0	2	12	12	20	5	CT	No CT

10	49	1c (m)	1a (1/6sn)	0	0	2	12	12	15	8	No CT	**CT**

2	49	1c	1mi (2/16)	0	0	2	12	12	10	9	CT	No CT

16	39	2	0 (0/3sn)	0	0	2	12	9	10	25	CT	No CT

20	71	2	1a (1/12)	1	1	3	12	12	15	21	CT	No CT

45	50	1c	1a (1/10)	1	0	2	8	12	18	20	CT	No CT

12	69	1c	0 (0/5sn)	0	0	3	12	12	25	31	No CT	**CT**

38	64	2	0 (0/2sn)	0	0	3	12	0	50	37	No CT	**CT**

## Data Availability

The data were analyzed from clinical reports which were documented in the information system of the Breast Cancer Center of the Leopoldina Hospital Schweinfurt.
